# Characterization of *Vibrio parahaemolyticus* isolated from stool specimens of diarrhea patients in Nantong, Jiangsu, China during 2018–2020

**DOI:** 10.1371/journal.pone.0273700

**Published:** 2022-08-26

**Authors:** Junfang Sun, Xue Li, Zimeng Hu, Xingfan Xue, Miaomiao Zhang, Qimin Wu, Wei Zhang, Yiquan Zhang, Renfei Lu

**Affiliations:** 1 Department of Clinical Laboratory, Affiliated Nantong Hospital 3 of Nantong University, Nantong, Jiangsu, China; 2 School of Animal Medicine, Nanjing Agricultural University, Nanjing, Jiangsu, China; 3 School of Medicine, Jiangsu University, Zhenjiang, Jiangsu, China; Nitte University, INDIA

## Abstract

*Vibrio parahaemolyticus* is the leading cause of acute seafood-associated gastroenteritis worldwide. The aim of this study was to investigate the presence of virulence genes, biofilm formation, motor capacities and antimicrobial resistance profile of *V*. *parahaemolyticus* isolates isolated from clinical samples in Nantong during 2018–2020. Sixty-six *V*. *parahaemolyticus* strains isolated from stool specimens of diarrheal patients were examined. The PCR results showed that there were two *tdh*^+^*trh*^+^ isolates, four *tdh*^-^*trh*^-^ isolates and sixty *tdh*^+^*trh*^-^ isolates, accounting for 3.0%, 6.1% and 90.9%, respectively. All the *tdh* carrying isolates manifested the positive reactions for the Kanagawa phenomenon (KP) test. Most of the isolates harbored at least one of the specific DNA markers of ‘pandemic group’ strains, suggesting that the dominant isolates of *V*. *parahaemolyticus* in Nantong might belong to the new O3: K6 or its serovariants. All *tdh*^+^ isolates possessed the Vp-PAI genes, but no *tdh*^-^*trh*^-^ isolates carried the T3SS2 genes. All isolates were biofilm producers and had relatively strong motor capacities. In addition, the *V*. *parahaemolyticus* isolates were resistant to ampicillin (98.5%), cefuroxime (75.6%), cefepime (66.7%), piperacillin (59.1%) and ampicillin/sulbactam (50.0%), but sensitive to ciprofloxacin (100.0%), levofloxacin (100.0%), trimethoprim-sulfamethoxazole (98.5%), gentamicin (98.5%), amikacin (97%), meropenem (71.2%), and ceftazidime (56.1%). Multidrug-resistant isolates in clinical might be related to the inappropriate use of antimicrobials in aquaculture.

## Introduction

*Vibrio parahaemolyticus*, a Gram-negative, highly motile, halophilic bacterium, is naturally found in marine ecosystems [1]. This bacterium is the leading cause of seafood-associated gastroenteritis in many countries including China [2–5]. Human infections with *V*. *parahaemolyticus* are usually caused by consumption of raw or undercooked seafood [6]. Pathogenic isolates usually produce thermostable direct hemolysin (TDH; encoded by *tdh*) and/or TDH-related hemolysin (TRH; encoded by *trh*) [7]. However, other factors such as the type III secretion systems (T3SS1 and T3SS2), urease (encoded by *ure*) and proteases also play roles in the pathogenesis of *V*. *parahaemolyticus* [6, 7]. T3SS1 is expressed by both pathogenic and non-pathogenic isolates, whereas T3SS2 only exists in pathogenic isolates [8]. The T3SS2 gene cluster and the two copies of *tdh* genes are present in a pathogenicity island known as Vp-PAI located on the smaller chromosome 2 of *V*. *parahaemolyticus* [9]. *V*. *parahaemolyticus* can utilize T3SS2 to efficiently inject TDH into target cells as an effector that contributes to intestinal fluid accumulation in an animal model [10].

There are total 13 somatic (O) antigens and 71 capsular (K) antigens in *V*. *parahaemolyticus* making up more than 70 serotypes [11]. However, since 1996, the new O3: K6 and its serovariants (O4: K68, O1: K25, O1: KUT, O1: K26 etc.) known as the ‘pandemic group’ had accounted for the majority of clinical isolates [12]. The ‘pandemic group’ isolates usually carried the *tdh* gene but not the *trh* and *ure* genes [12]. *V*. *parahaemolyticus* can be confirmed by the species-specific thermolabile hemolysin (*tlh*) and *toxR* genes [13–16], while the ‘pandemic group’ isolates can be distinguished by PCR targeting on several specific DNA markers, including the group-specific (GS) DNA sequence of *toxRS*/*new* [17], the ORF8 located on the f237 phage [18], the insertion sequence in the ORF of HU-α [19], the pandemic group specific (PGS) sequence [20], and the DNA fragment of VP2905 ORF [21].

The increasing number of *V*. *parahaemolyticus* isolates is shown to be resistant to multiple antibiotics due to inappropriate use of antimicrobials in aquaculture [15, 22–25]. In particular, the emergence of multi-drug resistant isolates should be given sufficient attention. *V*. *parahaemolyticus* isolates harboring the class 1 integrons of *dfrA14*-*bla*_VEB-1_-*aadB* and *bla*_VEB-1_-*aadB*-*arr2*-*cmlA*-*bla*_OXA-10_-*aadA1*, which are strongly associated with multi-drug resistance to various antibiotics including ampicillin, ceftazidime, cefotaxime and gentamicin, have been isolated from ready-to-eat foods in China [26]. Biofilms are extracellular matrix-enclosed bacterial colonies on surfaces [27]. *V*. *parahaemolyticus* is able to form biofilms on seafood surfaces, which enhance resistance to adverse growth conditions and/or chemical agents such as detergents and antibiotics thereby improving the survival rate and pathogenicity of the bacteria [27]. The biofilm formation ability of *V*. *parahaemolyticus* requires some specific genes, such as those associated with the biosynthesis of flagella, pili and exopolysaccharide [27, 28].

Nantong is located in the southeast of Jiangsu, bordering the Yellow Sea, with a coastline of over 200 km. The threat of *V*. *parahaemolyticus* to the health of citizens should be given adequate attention with the increasing of seafood consumption. Nevertheless, there is limited literature involving the prevalence or pathogenic profiles of *V*. *parahaemolyticus* in this city. In this study, a total of 66 *V*. *parahaemolyticus* isolates were isolated from stool specimens of diarrhoeal cases in Nantong, Jiangsu, China during 2018–2020. The polymerase chain reaction (PCR) assay was applied to screen the virulence-associated genes including *tdh*, *trh*, *ure*, *Mtase* and Vp-PAI genes (*vopP*, *vscC2*, *vopC* and VPA1376), as well as the species-specific marker genes *tlh* and *toxR*. All the isolates were subjected for screening of pandemic genotype by detecting the presence of *PGS* sequence (PGS-PCR), *toxRS*/*new* (GS-PCR), HU-α and *orf8*. At the same, a series of phenotypic experiments were employed to detect the hemolytic activities, biofilm formation abilities, motor (swimming and swarming) capacities and antimicrobial resistance profile of the *V*. *parahaemolyticus* isolates.

## Materials and methods

### Isolation of *V*. *parahaemolyticus*

Stool specimens from diarrhoeal cases (watery or loose stools with a duration of no more than 7 days) admitted in the different hospitals in Nantong were collected during 2018–2020, and screened for the presence of *V*. *parahaemolyticus* by applying the published methods [25, 29]. Briefly, stool specimens were inoculated into 5 ml of Alkaline Peptone Water (APW) (Polypeptone 10 g/L; Sodium chloride 10 g/L; pH8.6) and incubated at 37°C with shaking for 12 h. The APW-enriched culture was diluted 10,000-fold with the phosphate-buffered saline (PBS), and then 200 μL of the diluted samples were spread onto Thiosulphate Citrate Bile Salts Sucrose (TCBS; Beijing Land Bridge, China) agar plate, and incubated at 37°C for 12 h. The green or blue-green colonies were selected as presumed *V*. *parahaemolyticus* and then characterized by VITEK automatic biochemical analyzer (bioMerieux, France).

Ethics approval was not requested because no human or animal subjects were involved.

### Polymerase chain reaction (PCR) assay

Approximately 20 μL glycerol stock of *V*. *parahaemolyticus* was inoculated into 5 mL 2.5% Bacto heart infusion (HI; BD Bioscience, USA) broth supplemented with 1.5% (w/v) NaCl and incubated at 37°C with shaking at 200 rpm for 12 h, followed by centrifugation at 8000 g for 5 min. The genomic DNA was isolated using a QIAamp DNA mini Kit (Qiagen, Germany), and the concentration of DNA was determined by a NanoDrop spectrophotometry (ThermoFisher Scientific, USA).

Primers for PCR were synthesized by GRNEWIZ (Suzhou, China) and listed in Table 1. The PCR reaction mixture contained 10 μL 2×*Taq* PCR Mastermix (TIANGEN BIOTECH CO., LTD., China), 2 μL genomic DNA (10 ng/μL), 1 μL primer pair solution (10 μM each), and 7 μL sterile distilled water. PCR amplification was performed as the following conditions: pre-denaturation at 95°C for 5 min, followed by 30 cycles of denaturation at 94°C for 50 s, annealing at 54°C for 50 s, and extension at 72°C for 50 s, and ending extension at 72°C for 5 min. PCR products were detected by 1% agarose gel electrophoresis.

**Table 1:** Primers used in this study.

Target	Sequence (forward/reverse, 5ꞌ→3ꞌ)	Amplicon size (bp)	Reference
*toxR*/*new*	FTAATGAGGTAGAAACA/ACGTAACGGGCCTACA	651	[25]
*PGS* sequence	TTCGTTTCGCGCCACAACT/TGCGGTGATTATTCGCGTCT	235	[25]
*Mtase*	GTCTTGTCGAATAGAACTCTGA/TAAGCTCCAAAATCCATACG	683	[25]
*tlh*	AAAGCGGATTATGCAGAAGCACTG/GCTACTTTCTAGCATTTTCTCTGC	450	[25]
*tdh*	GTAAAGGTCTCTGACTTTTGGAC/TGGAATAGAACCTTCATCTTCACC	269	[25]
*trh*	TTGGCTTCGATATTTTCAGTATCT/CATAACAAACATATGCCCATTTCCG	500	[25]
*vopC*	CAGAGTTGGTTTCGCAG/CTGGTACGCCTCTTGGACAG	579	[25]
*vopP*	CGTCCAACTCTATTGTTGTG/CAATGTTGGCTATTCGGTTG	393	[25]
*vscC2*	GCGGTCTATTGCTATCCT/TCTTGGTATTGATAGTGGGTG	362	[25]
VPA1376	GCTCTCCTTGGTACCAATCAC/CTGGGATCTTGATGTCAAGGT	1067	[25]
HU-a	CGATAACCTATGAGAAGGGAAACC/CTAGAAGGAAGAATTGATTGTCAAATAATG	474	[25]
*ure*	CTTGTCATCGGGTGTCACTA/GATGTTAGGTTCACCTACTGACT	464	[25]
*orf8*	GTTCGCATACAGTTGAGG/AAGTACACAGGAGTGAG	700	[25]
*toxR*	GTCTTCTGACGCAATCGTTG/ATACGAGTGGTTGCTGTCATG	368	This study

### Biofilm crystal violet (CV) staining

CV staining was performed as previously described [30]. Briefly, the overnight cultures were diluted 50-fold into 5 mL HI broth and cultured at 37°C with shaking at 200 rpm to OD_600_ equals to 1.4. The resultant cultures were 50-fold diluted into 2 mL Difco marine (M) broth 2216 (BD Biosciences, USA) in 96-well plates (Corning Inc., Untied States) and allowed to grow at 30°C with shaking at 150 rpm for 48 h. The surface attached biofilms *in vitro* were stained with 0.1% CV. The bound CV was dissolved with 20% ethanol, and the OD_570_ values were then determined as the index of CV staining.

### Swimming motility

Swimming motility assay was performed as previously described [31]. Briefly, the overnight cell cultures were diluted 50-fold into 5 mL HI broth and cultured at 37°C with shaking at 200 rpm to OD_600_ equals to 1.4. Thereafter, 2 μL of the culture was inoculated into the semi-solid swim plates (1% Oxoid Tryptone, 2% NaCl [Merck, Germany], and 0.2% Difco Noble agar [BD Biosciences, USA]). Diameter of swimming area was measured after incubation at 37°C for 2 h.

### Swarming motility

Swarming motility assay was performed as previously described [31]. Briefly, the overnight cell cultures were diluted 50-fold into 5 mL HI broth and cultured at 37°C with shaking at 200 rpm to OD_600_ equals to 1.4. Thereafter, 2 μL of the culture was spotted on the swarm plate (2.5% Bacto heart infusion, 1.5% NaCl, and 1.8% Difco noble agar). Diameter of swarming zone was measured after incubation at 37°C for 48 h.

### Kanagawa phenomenon (KP) test

KP test was performed as previously described [32]. Briefly, 5 μL of the overnight cell culture was inoculated onto Wagatsuma agar (CHROMagar, China) containing 5% rabbit red blood cells (RBCs). Isolates with β-hemolysis after incubation at 37°C were considered as the KP positive.

### Antibiotic susceptibility testing (AST)

The VITEK 2 AST-GN09 antimicrobial sensitivity kit contains the following antimicrobial agents: ampicillin (AMP), ampicillin/sulbactam (SAM), piperacillin (PIP), piperacillin/tazobactam (TZP), cefazolin (CZ), cefuroxime (CXM), ceftazidime (CAZ), cefepime (FEP), meropenem (MEM), amikacin (AN), gentamicin (CN), ciprofloxacin (CIP), levofloxacin (LEV), and trimethoprim-sulfamethoxazole (SXT). A proper amount of separated and purified bacteria was added into a test tube containing 3 mL 0.45% NaCl solution, adjusting the turbidity of the bacteria solution to be the same as that of 0.5–0.63 Macmillan tube, taking 145 μL of 0.5–0.63 Macmillan unit bacteria suspension in a testing tube. AST for *V*. *parahaemolyticus* isolates was determined by minimum inhibitory concentrations (MICs) using a VITEK2 Compact automatic microbial analyzer (bioMérieux, France) [33]. The results were categorized as resistant (R), intermediate (I), or susceptible (S).

### Replicates and statistical methods

PCR, KP test and AST were performed two times with the same results. The swimming, swarming and CV staining were performed three independent bacterial cultures with three replicates for each, and the results were expressed as the mean ± standard deviation (SD). Paired Student’s *t*-tests were employed to calculate the statistical significance. *P* < 0.01 was considered as the significant.

## Results

### Identification of virulence genes in clinical *V*. *parahaemolyticus* isolates

A total of 66 isolates were isolated from stool specimens. All the isolates were confirmed by the VITEK automatic biochemical analysis. There were two *tdh*^+^*trh*^+^ isolates, four *tdh*^-^*trh*^-^ isolates and sixty *tdh*^+^*trh*^-^ isolates (Table 2), accounting for 3.0%, 6.1% and 90.9%, respectively. No isolate was *tdh*^-^*trh*^+^. The *tlh* and *toxR* genes were detected in all isolates (Table 2). The *toxR*/*new*, *orf8* and HU-α genes were only detected in the *tdh*^+^*trh*^-^ isolates (Table 2), and the prevalence of these genes was all 40.9% (27/66). The prevalence of *PGS* sequence was 100.0% (2/2) in *tdh*^+^*trh*^+^ isolates, 86.7% (52/60) in *tdh*^+^*trh*^-^ isolates and 50.0% (2/4) in *tdh*^-^*trh*^-^ isolates (Table 2). The prevalence of *ure* was 100.0% (2/2) in *tdh*^+^*trh*^+^ isolates, 0.0% (0/60) in *tdh*^+^*trh*^-^ isolates and 25.0% (1/4) in *tdh*^-^*trh*^-^ isolates (Table 2). The prevalence of *Mtase* was 0.0% (0/2) in *tdh*^+^*trh*^+^ isolates, 45.0% (27/60) in *tdh*^+^*trh*^-^ isolates and 25.0% (1/4) in *tdh*^-^*trh*^-^ isolates (Table 2). The other four virulence genes, *vopP* (100.0%; Table 2), *vscC2* (100.0%; Table 2), *vopC* (98.3%; Table 2), and VPA1376 (98.3%; Table 2), were detected in the genomic DNA of *tdh*^+^*trh*^-^ isolates. One *tdh*^-^*trh*^-^ isolate was also confirmed to harbor the VPA1376 gene (Table 2).

**Table 2:** Presence of virulence genes in the 66 clinical *V*. *parahaemolyticus* isolates.

Strain ID	*tlh*	*tdh*	*trh*	*toxR*/*new*	*PGS* sequence	*toxR*	*ure*	*MTase*	*orf8*	HU-α	*vopP*	*vscC2*	*vopC*	VPA1376
VP5	+	+	+	**-**	+	+	+	-	-	-	-	-	-	-
VP19	+	+	+	-	+	+	+	-	-	-	-	-	-	-
VP2	+	+	-	-	+	+	-	-	-	-	+	+	+	+
VP3	+	+	-	-	+	+	-	-	-	-	+	+	+	+
VP4	+	+	-	-	+	+	-	-	-	-	+	+	+	+
VP6	+	+	-	-	+	+	-	-	-	-	+	+	+	+
VP8	+	+	-	-	+	+	-	-	-	-	+	+	+	+
VP9	+	+	-	-	+	+	-	-	-	-	+	+	+	+
VP10	+	+	-	-	+	+	-	-	-	-	+	+	+	+
VP11	+	+	-	-	+	+	-	-	-	-	+	+	+	+
VP12	+	+	-	-	-	+	-	-	-	-	+	+	+	+
VP13	+	+	-	+	+	+	-	+	+	+	+	+	+	+
VP14	+	+	-	-	+	+	-	-	-	-	+	+	+	+
VP16	+	+	-	+	-	+	-	+	+	+	+	+	+	+
VP17	+	+	-	+	-	+	-	+	+	+	+	+	+	+
VP18	+	+	-	+	+	+	-	+	+	+	+	+	+	+
VP20	+	+	-	+	+	+	-	+	+	+	+	+	+	+
VP29	+	+	-	-	+	+	-	-	-	-	+	+	+	+
VP30	+	+	-	-	+	+	-	-	-	-	+	+	+	+
VP36	+	+	-	+	+	+	-	+	+	+	+	+	+	+
VP37	+	+	-	-	+	+	-	-	-	-	+	+	+	+
VP39	+	+	-	-	-	+	-	-	-	-	+	+	+	+
VP40	+	+	-	-	+	+	-	-	-	-	+	+	+	+
VP41	+	+	-	-	+	+	-	-	-	-	+	+	+	+
VP42	+	+	-	-	+	+	-	-	-	-	+	+	+	+
VP43	+	+	-	-	+	+	-	-	-	-	+	+	+	+
VP44	+	+	-	-	+	+	-	-	-	-	+	+	+	+
VP45	+	+	-	-	+	+	-	-	-	-	+	+	+	+
VP46	+	+	-	-	+	+	-	-	-	-	+	+	+	+
VP47	+	+	-	-	+	+	-	-	-	-	+	+	+	+
VP48	+	+	-	-	-	+	-	-	-	-	+	+	+	+
VP49	+	+	-	-	+	+	-	-	-	-	+	+	+	+
VP50	+	+	-	-	+	+	-	-	-	-	+	+	+	+
VP51	+	+	-	-	+	+	-	-	-	-	+	+	+	+
VP52	+	+	-	-	+	+	-	-	-	-	+	+	+	+
VP53	+	+	-	-	+	+	-	-	-	-	+	+	+	+
VP54	+	+	-	-	+	+	-	-	-	-	+	+	+	+
VP55	+	+	-	-	-	+	-	-	-	-	+	+	+	+
VP56	+	+	-	+	+	+	-	+	+	+	+	+	+	+
VP57	+	+	-	+	+	+	-	+	+	+	+	+	+	+
VP58	+	+	-	+	+	+	-	+	+	+	+	+	+	+
VP59	+	+	-	+	+	+	-	+	+	+	+	+	+	+
VP60	+	+	-	+	+	+	-	+	+	+	+	+	+	+
VP61	+	+	-	+	+	+	-	+	+	+	+	+	+	+
VP62	+	+	-	-	+	+	-	-	-	-	+	+	+	+
VP63	+	+	-	+	+	+	-	+	+	+	+	+	+	+
VP64	+	+	-	+	+	+	-	+	+	+	+	+	+	+
VP65	+	+	-	+	+	+	-	+	+	+	+	+	+	+
VP66	+	+	-	+	+	+	-	+	+	+	+	+	+	+
VP67	+	+	-	+	+	+	-	+	+	+	+	+	+	+
VP69	+	+	-	+	+	+	-	+	+	+	+	+	+	+
VP70	+	+	-	+	+	+	-	+	+	+	+	+	+	+
VP71	+	+	-	+	+	+	-	+	+	+	+	+	+	+
VP72	+	+	-	+	+	+	-	+	+	+	+	+	+	+
VP73	+	+	-	+	+	+	-	+	+	+	+	+	+	+
VP74	+	+	-	+	+	+	-	+	+	+	+	+	+	+
VP75	+	+	-	+	+	+	-	+	+	+	+	+	+	+
VP76	+	+	-	+	+	+	-	+	+	+	+	+	+	+
VP77	+	+	-	+	+	+	-	+	+	+	+	+	+	+
VP78	+	+	-	-	+	+	-	+	+	+	+	+	+	-
VP79	+	+	-	-	-	+	-	-	-	-	+	+	+	+
VP80	+	+	-	-	-	+	-	-	-	-	+	+	-	+
VP7	+	-	-	-	-	+	-	-	-	-	-	-	-	-
VP15	+	-	-	-	+	+	-	-	-	-	-	-	-	-
VP35	+	-	-	-	+	+	-	-	-	-	-	-	-	-
VP68	+	-	-	-	-	+	+	+	-	-	-	-	-	+

### Hemolytic activity of clinical *V*. *parahaemolyticus* isolates

**Fig 1:**
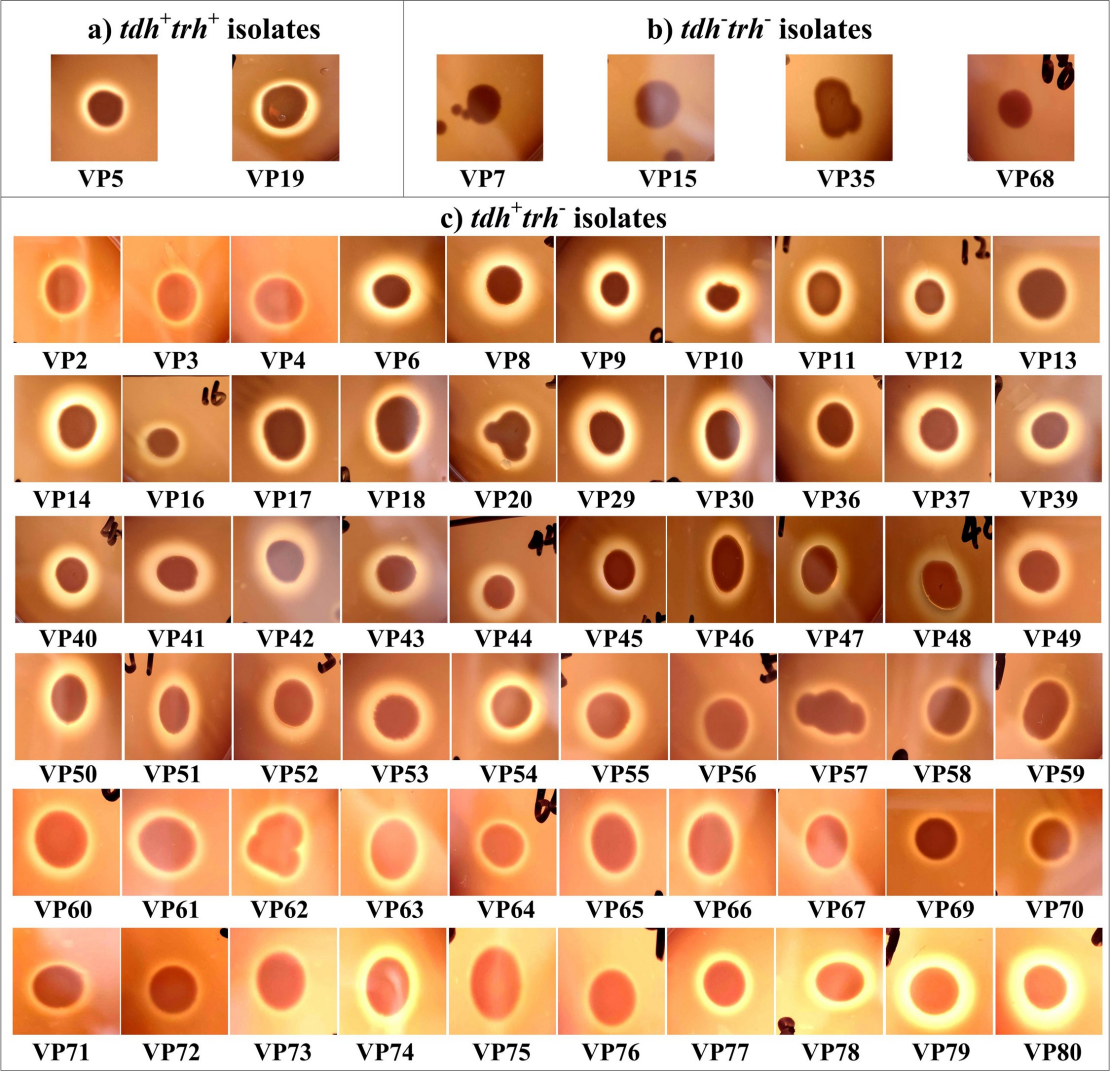
The hemolytic activity of *V*. *parahaemolyticus* isolates against RBCs was evaluated by observing whether there was a β-hemolysis zone surrounding the spot of growth on the Wagatsuma agar plate. The pictures shown here are representative images of *V*. *parahaemolyticus* cells on Wagatsuma agar.

The hemolytic activity of each isolate was measured by the KP test on the Wagatsuma agar supplemented with 5% RBCs. As shown in Fig 1, all the *tdh*^+^*trh*^*+*^ and *tdh*^+^*trh*^-^ isolates were recorded as positive reactions with a β hemolysis zone surrounding the growth spot, whereas all the *tdh*^-^*trh*^-^ isolates gave negative reactions. These results suggested that all isolates harboring the *tdh* gene was able to express active TDH.

### Biofilm formation by clinical *V*. *parahaemolyticus* isolates

Biofilm formation by the 66 isolates was investigated by the CV staining. As shown in Table 3, all the isolates were biofilm producers. Regarding the degrees of biofilm [34], 50.0% of *tdh*^+^*trh*^+^ isolates and 10.0% of *tdh*^+^*trh*^-^ isolates were weak producers, 50.0% of *tdh*^+^*trh*^+^ isolates, 48.3% of *tdh*^+^*trh*^-^ isolates and 100% of *tdh*^-^*trh*^-^ isolates were moderate producers, while 41.7% of *tdh*^+^*trh*^-^ isolates were strong producers.

**Table 3:** Biofilm formation by *V*. *parahaemolyticus* isolates at 30°C.

Isolates	Total No.	Degree of biofilm formation (%, average OD ± SD)			Overall biofilm producers
		Weak	Moderate	Strong	
***tdh*** ^**+**^ ***trh*** ^**+**^	2	1 (50.0%, 0.197 ± 0.022)	1 (50.0%, 0.532 ± 0.051)	0	2 (100.0%)
***tdh*** ^**+**^ ***trh*** ^**-**^	60	6 (10.0%, 0.167 ± 0.017)	29 (48.3%, 0.459 ± 0.086)	25 (41.7%, 1.381 ± 0.966)	60 (100.0%)
***tdh*** ^**-**^ ***trh*** ^**-**^	4	0	4 (100.0%, 0.401 ± 0.056)	0	4 (100.0%)

### Swimming and swarming motility of clinical *V*. *parahaemolyticus* isolates

*V*. *parahaemolyticus* possesses dual flagellar systems, i.e., a single polar flagellum for swimming in liquid and peritrichous lateral flagella for swarming on surfaces [35]. In this study, the swimming and swarming capacities were compared between each clinical isolates and the reference strain RIMD2210633. According to this, the motor abilities of clinical isolates were divided into three grades: weak, medium, and strong, which respectively indicated that their motor abilities were much lower, no difference with, or significantly higher than those of RIMD2210633. As shown in Table 4, all the isolates were swimmers; 11.7% of *tdh*^+^*trh*^-^ isolates and 50.0% of *tdh*^-^*trh*^-^ isolates were weak swimmers; 50.0% of *tdh*^+^*trh*^+^ isolates and 25.0% of *tdh*^+^*trh*^-^ isolates were moderate swimmers, while 50.0% of *tdh*^+^*trh*^+^ isolates, 63.3% of *tdh*^+^*trh*^-^ isolates and 50.0% of *tdh*^-^*trh*^-^ isolates were strong swimmers. Similarly, all of the isolates were swarm cells (Table 5), among which 100% of *tdh*^+^*trh*^-^ isolates, 20.0% of *tdh*^+^*trh*^-^ isolates and 50.0% of *tdh*^-^*trh*^-^ isolates were moderate swarm cells; 80.0% of *tdh*^+^*trh*^-^ isolates and 50.0% of *tdh*^-^*trh*^-^ isolates were strong swarm cells. These results indicated that all the isolates had a relatively strong motor capacity.

**Table 4:** Swimming motility of *V*. *parahaemolyticus* isolates.

Isolates	Total No.	Degree of swimming ability (%, average mm ± SD)			Overall swimming producers
		Weak	Moderate	Strong	
***tdh*** ^**+**^ ***trh*** ^**+**^	2	0	1 (50.0%, 7.000 ± 1.000)	1 (50.0%, 10.667* ± 0.577)	2 (100.0%)
***tdh*** ^**+**^ ***trh*** ^**-**^	60	7 (11.7%, 3.405* ± 0.443)	15 (25.0%, 6.233 ± 0.793)	38 (63.3%, 10.550* ± 0.820)	60 (100.0%)
***tdh*** ^**-**^ ***trh*** ^**-**^	4	2 (50.0%, 3.750* ± 0.683)	0	2 (50.0%, 8.833* ± 0.382)	4 (100.0%)
**RIMD2210633**		6.500 ± 0.500			

**Table 5:** Swarming motility of *V*. *parahaemolyticus* isolates.

Isolates	Total No.	Degree of swarming ability (%, average mm ± SD)			Overall swarming producers
		Weak	Moderate	Strong	
***tdh*** ^**+**^ ***trh*** ^**+**^	2	0	2 (100%, 14.417 ± 0.382)	0	2 (100.0%)
***tdh*** ^**+**^ ***trh*** ^**-**^	60	0	12 (20.0%, 14.417 ± 0.458)	48 (80.0%, 16.799* ± 0.675)	60 (100.0%)
***tdh*** ^**-**^ ***trh*** ^**-**^	4	0	2 (50.0%, 13.750 ± 0.433)	2 (50.0%, 17.833* ± 0.866)	4 (100.0%)
**RIMD2210633**		14.167 ± 0.289			

### Antibiotic susceptibility of clinical *V*. *parahaemolyticus* isolates

AST was performed on clinical *V*. *parahaemolyticus* isolates using 14 antibiotics. As shown in Table 6, the *V*. *parahaemolyticus* isolates were extremely resistant to ampicillin (98.5%), followed by cefuroxime (75.6%), cefepime (66.7%), piperacillin (59.1%), ampicillin/sulbactam (50.0%), piperacillin/tazobactam (45.5%), ceftazidime (43.9%), cefazolin (28.8%), and meropenem (28.8%). All the isolates were sensitive to ciprofloxacin (100.0%) and levofloxacin (100.0%), followed by trimethoprim-sulfamethoxazole (98.5%), gentamicin (98.5%), amikacin (97.0%), meropenem (71.2%), ceftazidime (56.1%), piperacillin/tazobactam (40.9%), piperacillin (36.4%), and ampicillin/sulbactam (28.8%).

**Table 6:** Antibiotics resistance profiles of clinical *V*. *parahaemolyticus* isolates.

Antibiotics	Number (%) of S	Number (%) of I	Number (%) of R
Ampicillin	1 (1.5)	0 (0.0)	65 (98.5)
Ampicillin/sulbactam	19 (28.8)	14 (21.2)	33 (50.0)
Piperacillin	24 (36.4)	3 (4.5)	39 (59.1)
Piperacillin/tazobactam	27 (40.9)	9 (13.6)	30 (45.5)
Cefazolin	2 (3.0)	45 (68.2)	19 (28.8)
Cefuroxime	0 (0.0)	16 (24.2)	50 (75.6)
Ceftazidime	37 (56.1)	0 (0.0)	29 (43.9)
Cefepime	22 (3.3)	0 (0.0)	44 (66.7)
Meropenem	47 (71.2)	0 (0.0)	19 (28.8)
Amikacin	64 (97.0)	2 (3.0)	0 (0.0)
Gentamicin	65 (98.5)	1 (1.5)	0 (0.0)
Ciprofloxacin	66 (100.0)	0 (0.0)	0 (0.0)
Levofloxacin	66 (100.0)	0 (0.0)	0 (0.0)
trimethoprim-sulfamethoxazole	65 (98.5)	0 (0.0)	1 (1.5)

## Discussion

*V*. *parahaemolyticus* can be easily isolated from seawater and seafood [36–39]. However, most of environmental isolates are non-pathogenic with a very low detection rate of the *tdh* and/or *trh* genes [14, 15, 29, 38–41]. By contrast, majority of clinical isolates harbor the *tdh* and/or *trh* genes [14, 15, 29, 40, 41]. In this study, 66 *V*. *parahaemolyticus* isolates were isolated from stool specimens, of these, 62 isolates had the *tdh* gene, and 2 isolates simultaneously contained the *trh* gene. The proportion of clinical isolates containing the *tdh* and/or *trh* genes is similar to the results of other researchers [15, 39, 42–44]. Significantly, four isolates harbored neither the *tdh* nor the *trh* gene but had the ability to cause disease, which has been similarly reported in previous studies [13, 43]. The pathogenic mechanisms of clinical isolates carrying neither *tdh* nor *trh* still need to be further investigated.

The *tlh* and *toxR* genes are the species-specific markers that can be detected in all the *V*. *parahaemolyticus* isolates [13–16]. The *PGS* sequence, *toxR*/*new*, *orf8* and HU-α genes were used as specific DNA markers to distinguish the ‘pandemic group’ isolates from other serotypes [17–20]. The data showed that most of the isolates harbor one or more specific DNA markers of the ‘pandemic group’, indicating that the dominant isolates of *V*. *parahaemolyticus* in Nantong might belong to the new O3: K6 or its serovariants.

The ability to product urease by *V*. *parahaemolyticus* has been demonstrated highly correlates with the existing of the *trh* gene [45]. As shown in this study, all the *trh* positive isolates possessed the *ure* gene. However, one *tdh*^-^*trh*^-^ isolate also harbored the *ure* gene. The presence of *ure* in *tdh*^-^*trh*^-^ isolate might be due to the presence of *trh* gene variant that could not be detected by the PCR used in this study. In addition, the *MTase* gene encoding a putative virulence-associated DNA methyltransferase was major detected in the *tdh*^+^*tdh*^-^ isolates, which was similar to a previous report [46]. T3SS1 and T3SS2 are also thought to be involved in the pathogenicity of *V*. *parahaemolyticus* [47]. T3SS2 was only present in the *tdh*^+^ isolates [9], but a novel T3SS2 belonging to a different lineage was also detected in the *trh*^+^ isolates [48]. In this work, we showed that all the *tdh*^+^ isolates possessed at least two of the *vopP*, *vscC2*, *vopC* and VPA1376 genes located in the Vp-PAI gene cluster (T3SS2). None of the T3SS2 genes (*vopP*, *vscC2* and *vopC*) were detected in the *tdh*^-^*trh*^-^ isolates, but one of the isolates harbored the VPA1376 gene, suggesting this gene was likely to be acquired by horizontal transfer.

The antimicrobial resistance of *V*. *parahaemolyticus* has become one of the most serious threats to fish farming, food safety and public health. Most of the isolates in this study exhibited a high level of resistance to ampicillin, cefuroxime, cefepime, piperacillin, and ampicillin/sulbactam, but sensitive to ciprofloxacin, levofloxacin, trimethoprim-sulfamethoxazole, gentamicin, amikacin, meropenem, and ceftazidime. *V*. *parahaemolyticus* isolates are universally resistant to ampicillin according to literatures [3, 15, 24, 25, 40, 41, 44, 49–53]. The *bla*_CARB-17_ gene encoding a novel class A carbenicillin-hydrolyzing β-lactamase family of β-lactamase that is responsible for the resistance to penicillin was detected in all tested *V*. *parahaemolyticus* isolates [54]. However, the antimicrobial resistance profiles of *V*. *parahaemolyticus* might vary in different reports, for instance, 60.3% of *V*. *parahaemolyticus* isolates from rearing water samples of shrimp farms in Fujian, China exhibited resistance to gentamicin in the report of Shu Zhao, et al.[50], and 50.8% and 47.6% of isolates from African salad samples in Nigeria were resistant to amikacin and ceftazidime in the report of Etinosa O. Igbinosa, et al. [3]. No matter how different of the antimicrobial resistance profiles, emergence of multi-drug resistant *V*. *parahaemolyticus* is a serious threat to aquaculture and public health.

*V*. *parahaemolyticus* possesses the strong ability to form biofilms and persist on the surfaces of seafood for the long existence [27]. This study showed that all clinical *V*. *parahaemolyticus* isolates were biofilm producers. The ability to form biofilms is related to the source of isolates and cultural temperature, and pathogenic isolates produced more biofilms than non-pathogenic isolates [34, 55]. Incubation temperature of 37°C was considered as optimum temperature for biofilm formation by *V*. *parahaemolyticus* [56]. Importantly, it is universally acknowledged that bacterial cells in biofilms are much more resistant to adverse conditions than planktonic cells [27]. Therefore, the biofilm produced by *V*. *parahaemolyticus* hugely increases the potential risks to seafood consumers. The movements of *V*. *parahaemolyticus* propelled by flagella can be divided into swimming and swarming, both of which are required for the initial stages of biofilm formation [28]. The data showed that all *V*. *parahaemolyticus* isolates had relatively strong motor capacities, which were consistent with the observational facts that all the isolates were biofilm producers.

In conclusion, this study focused on the virulence, biofilm formation, motilities and antimicrobial resistance of *V*. *parahaemolyticus* isolates isolated from stool specimens of diarrheal cases in Nantong during 2018–2020. A total of 66 isolates were collected, 93.9% of them carried the *tdh* gene and manifested the positive reactions for KP test. Most of the isolates harbored at least one of the specific DNA markers of ‘pandemic group’ strains, suggesting that the dominant isolates of *V*. *parahaemolyticus* in Nantong belonged to the new O3: K6 and its serovariants. 100.0% of *tdh*^+^ isolates possessed the Vp-PAI genes, but only one *tdh*^-^*trh*^-^ isolate carried the T3SS2 gene. All *V*. *parahaemolyticus* isolates were biofilm producers and had relatively strong motor capacities. In addition, the *V*. *parahaemolyticus* isolates were resistant to ampicillin, cefuroxime, cefepime, piperacillin and ampicillin/sulbactam, but sensitive to ciprofloxacin, levofloxacin, trimethoprim-sulfamethoxazole, gentamicin, amikacin, meropenem and ceftazidime. The data presented here would be beneficial for preventing and controlling the seafood-associated illnesses caused by *V*. *parahaemolyticus* in Nantong, Jiangsu, China.
